# Single-dose of a replication-competent adenovirus-vectored vaccine provides sterilizing protection against Rift Valley fever virus challenge

**DOI:** 10.3389/fimmu.2022.907675

**Published:** 2022-11-11

**Authors:** Ting Bian, Busen Wang, Guangcheng Fu, Meng Hao, Yi Chen, Ting Fang, Shuling Liu, Changming Yu, Jianmin Li, Wei Chen

**Affiliations:** ^1^ Beijing Institute of Biotechnology, Academy of Military Medical Sciences, Beijing, China; ^2^ Frontier Biotechnology Laboratory, Zhejiang University-Hangzhou Global Scientific and Technological Innovation Center, Hangzhou, China

**Keywords:** RVFV vaccine, adenovirus serotype 4 vector, glycoproteins, replication-competent, single-dose immunization, sterilizing protection

## Abstract

Rift Valley fever virus (RVFV) is one of the most important virulent pathogens causing severe disease in animals and humans. However, there is currently no approved vaccine to prevent RVFV infection in humans. The use of human adenovirus serotype 4 (Ad4) as a vector for an RVFV vaccine has not been reported. Here, we report the generation of a replication-competent recombinant Ad4 vector expressing codon-optimized forms of the RVFV glycoproteins Gn and Gc (named Ad4-GnGc). Intramuscular immunization with Ad4-GnGc elicited robust neutralizing antibodies against RVFV and cellular immune responses in mice. A single low-dose vaccination with Ad4-GnGc completely protected interferon-α/β receptor-deficient A129 mice from lethal RVFV infection. More importantly, Ad4-GnGc efficacy was not affected by pre-existing immunity to adenovirus serotype 5, which currently exists widely in populations. These results suggest that Ad4-GnGc is a promising vaccine candidate against RVFV.

## Introduction

Emerging viral diseases can be unpredictable and may seriously impact human health and the global economy, as evidenced by the recent outbreaks of Ebola virus and the ongoing severe acute respiratory syndrome coronavirus-2 (SARS-CoV-2) pandemic. Rift valley fever virus (RVFV), identified in Kenya in 1930 (1, 2), is a mosquito-borne zoonotic infectious pathogen that poses a great threat to human and animal health with pandemic potential because of the global presence of its vectors and hosts (3–5). RVFV causes severe disease in livestock, especially ruminants, which results in abortion storms, as well as high fatality rates in newborn lambs and calves (6). Humans can be infected by being bitten by virus-carrying mosquitoes or through contact with the bodily fluids of infected animals (7). Most Rift Valley fever (RVF) patients display a self-limiting, acute, and febrile illness, while a small number of cases may progress to neurologic disorders, blindness, or lethal hemorrhagic fever (7, 8). RVF outbreaks mainly occur in sub-Saharan and North Africa, but in 2000, the first reported cases outside the African continent occurred in the Arabian Peninsula (9). Later, in 2016, China reported the first imported case of RVFV infection (10). Despite the significant impacts of RVFV on human health and the economy, there are no safe and efficacious prophylactic or therapeutic treatment options for human use.

Vaccination is one of the most economical and effective methods for prevention and control of RVF. However, there is no vaccine available for human use, and although several licensed veterinary vaccines are available (11), their respective shortcomings limit their widespread use (12). For example, the Smithburn, MP-12 and Clone 13 vaccines are all widely used live attenuated RVFV vaccines with strong immunogenicity, and a single immunization can induce long-lasting immunity in livestock, but they all carry certain risks of abortion and birth malformation in pregnant animals (12–14). In addition, like most live attenuated vaccines, they carry the risk of reversion to virulence. Formalin-inactivated RVFV vaccines, such as NDBR103 and TSI GSD 200, can be applied safely during pregnancy but require multiple boosters to achieve long-lasting immunity (12, 15), complicating their use in outbreak situations. For humans, only three vaccines have been evaluated in clinical trials: MP-12, TSI-GSD-200 and ChAdOx1 RVF. They all have a good safety profile in humans, but MP-12 still has a teratogenic risk in livestock, and TSI-GSD-200 requires multiple doses to achieve optimal efficacy (12). Although ChAdOx1 RVF has a low rate of pre-existing anti-vector immunity and shows good safety in animals, its ability to induce an immune response is significantly lower than that of a recombinant human adenovirus serotype 5 (Ad5) vector-based RVFV vaccine (16, 17). Due to their safety and ability to stimulate robust cellular and humoral immune responses in multiple species, recombinant adenoviruses, such as human adenovirus serotype 2 (Ad2), Ad5, human adenovirus serotype 26 (Ad26) and ChAdOx1, have been extensively explored as vaccine vectors for many pathogens (18–20). Among them, the Ad5 vector is the most widely used. At present, several vaccines based on this vector have entered clinical trials, targeting pathogens including Ebola virus (21), influenza virus (22) and SARS-CoV-2 (23). However, with the widespread application of recombinant Ad5-vectored vaccines, the phenomenon of pre-existing immunity against this vector is becoming increasingly common. Therefore, the identification of other adenoviral vectors with a low pre-existing immunity rate, high safety, good immunogenicity, and weak cross-reactivity with Ad5 is urgently needed. We therefore investigated whether recombinant human adenovirus serotype 4 (Ad4) could be used as another adenoviral vector for vaccine development. Live oral Ad4 has been approved for use as a vaccine against Ad4-induced respiratory disease by the military in the United States. Since beginning the use of this vaccine, the incidence of adenovirus-related diseases has been significantly reduced. The safety and efficacy of the vaccine have been well established by placebo-controlled clinical trials (24). A follow-up study also showed that this vaccine could induce long-term immunity (25). In addition, an Ad4-vectored influenza vaccine (Ad4-H5-Vtn) delivered to the upper respiratory tract could induce durable systemic and mucosal immunity and has entered clinical trials (26, 27), which further indicates that the Ad4 vector is a promising platform for vaccine development.

In this study, we generated a recombinant replication-competent Ad4-vectored vaccine, Ad4-GnGc, carrying codon-optimized genes encoding the Gn and Gc proteins of the RVFV MP-12 strain. We then investigated antibody and cell-mediated immune responses elicited by the vaccine, examined the effect of pre-existing Ad5 immunity on the efficacy of the vaccine, and finally demonstrated the protective efficacy of the candidate vaccine against RVFV challenge in a mouse model.

## Materials and methods

### Ethics statement

All animal experiments were performed in strict accordance with the Guide for the Care and Use of Laboratory Animals of the People’s Republic of China and approved by the Animal Care and Use Committee of Beijing Institute of Biotechnology, China (Permit number: IACUC-SWGCYJS-2021-006). Specific pathogen-free female BALB/c mice aged 6-8 weeks were obtained from SPF (Beijing) Biotechnology Co., Ltd. (Beijing, China), they were housed and bred in the animal facility of the Animal Center, Beijing Institute of Biotechnology. Interferon-α/β receptor-deficient A129 mice were preserved and housed in the animal facility of the Animal Center, Beijing Institute of Biotechnology. The animal experiments involving RVFV challenge were conducted in the animal biosafety level 2 (ABSL2) facilities of the Beijing Institute of Biotechnology.

### Cell lines and viruses

African green monkey kidney (Vero E6 cells) and human embryonic kidney (HEK293 cells) were cultured in Dulbecco’s modified Eagle’s medium (DMEM, Gibco) supplemented with 10% fetal bovine serum (FBS, Gibco), 100 U/mL penicillin, and 100 mg/mL streptomycin at 37°C and 5% CO_2_. The RVFV MP-12 strain and RVFV MP-12 strain expressing eGFP were rescued using a previously described method (28) and named rMP-12 and rMP-12-eGFP, respectively. The viruses were propagated in Vero E6 cells. The human Ad5 vector was constructed and preserved at the Beijing Institute of Biotechnology (21, 23, 29).

### Construction of the replication-competent Ad4-vectored vaccine expressing the RVFV glycoproteins Gn and Gc

The glycoproteins Gn and Gc of RVFV based on the MP-12 strain (accession number DQ380208.1) were codon optimized and synthesized. The tissue plasminogen activator (tPA) signal peptide was added to improve the expression of the antigenic proteins in mammalian cells. The genes were cloned into the pAd4-dE3 plasmid constructed by our laboratory to obtain pAd4-dE3-GnGc. Then, pAd4-dE3-GnGc was linearized and transfected into HEK293 cells using TurboFect transfection reagent (Thermo Fisher Scientific, USA) to rescue Ad4-GnGc. The transfected cells were passaged and collected until typical cytopathic effects were observed. The recombinant adenoviruses were then confirmed by sequencing and western blotting. Finally, the recombinant adenoviruses were propagated, purified by ion exchange chromatography and size exclusion, titrated, and stored at -80°C.

### Detection of Gn and Gc protein expression

To analyze the Ad4-GnGc-mediated expression of Gn and Gc, 1 × 10^6^ Vero E6 cells were infected with Ad4-GnGc (MOI=10). Forty-eight hours after infection, the cells were washed with phosphate-buffered saline (PBS) pH 7.4 and lysed with RIPA lysis buffer (Thermo Scientific, USA). Then, the supernatant was collected for sodium dodecyl sulfate–polyacrylamide gel electrophoresis (SDS–PAGE). Subsequently, the separated protein was transferred to polyvinylidene fluoride membranes by an eBlot L1 transfer system (GenScript, China) and incubated with an anti-Gn monoclonal antibody (at a final concentration of 0.5 µg/mL) or anti-Gc rabbit polyclonal antibody (1:3000 dilution). Corresponding secondary antibodies labeled with HRP were added at a final concentration of 0.1 µg/mL (Abcam, UK). The membranes were developed with a chemiluminescent substrate (Merck Millipore, USA), and images were acquired with an iBright 1500 imaging system (Thermo Fisher Scientific, USA). As an internal reference, β-actin was detected with an HRP-conjugated anti-β-actin antibody (Abcam, UK, 1:50000 dilution).

### Mouse vaccination and challenge experiments

BALB/c mice were immunized intramuscularly with 10^6^, 10^7^ or 10^8^ infectious units (IFU) of Ad4-GnGc or with PBS as a control on day 0. Sera were collected for Gn- and Gc-specific IgG and anti-RVFV neutralizing antibody (NAb) titration at different time points. Some mice immunized with the high dose of Ad4-GnGc or with PBS were euthanized on day 14 post-immunization for splenic cellular immune response detection.

For the pre-existing anti-vector immunity study, BALB/c mice were randomly divided into three groups (n = 12 in each group), and the first group was inoculated intramuscularly with 10^8^ IFU of Ad5 vector at week -4. At week 0, the first and second groups received injections of 10^8^ IFU of Ad4-GnGc, while the third group received PBS as a negative control. Sera were collected from vaccinated animals at different time points for antibody detection. Half of the mice in each group were euthanized on day 14 post-immunization for cellular immune response detection.

RVFV challenge experiments using an interferon-α/β receptor-deficient A129 mouse model were performed as previously described with slight modifications (30, 31). A129 mice (n = 5 in each group) immunized with a low dose (10^6^ IFU) or high dose (10^8^ IFU) of Ad4-GnGc or with PBS were challenged intraperitoneally with 2×10^4^ TCID_50_ of RVFV rMP-12 strain four weeks after vaccination. Considering that the survival rate of vaccine-immunized animals after challenge is a very important data to evaluate the protective effect of vaccines, in order to evaluate the protective ability of the candidate vaccine, we need to measure the survival rate of immunized mice and viral loads in livers and spleens after challenge, so we chose death as the endpoint. The mice were monitored and scored for clinical symptoms for 14 days as previously described with minor modifications (32). Briefly, rough hair (absent = 0, slightly = 1, markedly = 2), activity (normal = 0, slightly reduced = 1, reduced = 2, severely reduced = 3), eye discharge (absent = 0, slightly = 1, moderate = 2, severe = 3), and body weight (no reduced = 0, slightly reduced = 1, reduced = 2, severely reduced = 3). The final score was the addition of each individual score. The minimum score was 0 for healthy and 1-11 depending upon the severity. Animals that reached 8 points of score were euthanized. Animals that did not reach humane endpoint and survived the RVFV challenge were euthanized at the end of the experiment. Organ tissues were collected at necropsy for subsequent viral load studies, histopathology assays and immunohistochemical staining performed as described below. Animal experiments involving RVFV challenge were conducted in the ABSL2 facilities of the Academy of Military Medical Sciences, Beijing.

### Binding antibodies measured by ELISA

For measurement of RVFV Gn- and Gc-specific IgG titers in mice, 96-well microplates (Corning, USA) were coated with 100 µl of purified RVFV Gn or Gc at 2 µg/ml in carbonate–bicarbonate buffer (pH 9.6) at 4°C overnight. After washing three times with PBS containing 0.2% Tween-20 (PBST), the plates were blocked with 2% BSA in PBS at 37°C for 1 h. Serially diluted sera from mice were added to the plates and incubated at 37°C for 1 h. Then, the plates were washed and incubated with 1:20000 diluted HRP-conjugated goat anti-mouse IgG, IgG1, IgG2a (Abcam, UK) at 37°C for 1 h, followed by detection with a TMB substrate solution (Solarbio, China) at 450 nm/630 nm (SPECTRA MAX 190, Molecular Device, USA). The endpoint titers were defined as the reciprocal of the highest serum dilution that produced an optical absorbance value 2.1-fold higher than the optical absorbance value of the negative control.

### Focus reduction neutralization test

The neutralizing activity of sera from mice was assessed using a microneutralization assay, which was performed as previously described with some modifications (33, 34). In brief, serial dilutions of heat-inactivated mouse sera were incubated with 100 TCID_50_ of rMP-12-eGFP at 37°C for 1 h. Subsequently, 20000 Vero E6 cells were added to each well and incubated for 48 h. After infection, the cells were fixed with 4% formaldehyde for 2 h. Then, the cells were counterstained with DAPI (Thermo Fisher Scientific, 1:1000). Infected cells (eGFP) and total cells (DAPI) were quantified using a Celigo (Nexcelom) imaging cytometer. Infectivity was measured by assessing the accumulation of eGFP in Vero E6 cells. The FRNT_50_ was defined as the reciprocal of the serum dilution that exhibited 50% inhibition of viral infection compared to the level of infection in the virus-only well.

### Assessment of cellular immune responses

The cellular immune responses of vaccinated mice were assessed using mouse IFN-γ and IL-2 ELISpot Kits (MabTech, Sweden) according to the manufacturer’s protocols. A total of 1×10^5^ splenocytes from immunized mice were stimulated with fourteen peptides derived from the RVFV Gn and Gc proteins (5 μg/µl) identified previously (16) and seeded in precoated ELISpot plates for 16 h at 37°C with 5% CO_2_. The plates were washed five times with PBS and incubated with a biotin-conjugated detection antibody at room temperature for 1 h. After that, the plates were washed again and incubated with streptavidin-HRP at room temperature for 1 h. Then, the cells were washed again, and spots corresponding to antigen-specific cytokine-secreting cells were developed with TMB substrate for ELISpot. Finally, the plates were washed sufficiently with deionized water, and the spots were counted on an AID ELISPOT reader (AID GmbH, Strassberg, Germany).

Besides, the intracellular cytokine staining assay (ICCS) was also performed. 2×10^6^ mouse splenocytes were seeded in 24-well plates and stimulated with the fourteen peptides described above, together with BD GolgiStop™ to block cytokine secretion. After that, the splenocytes were blocked with anti-CD16/32 (S17011E clone, BioLegend, USA) and stained with a mixture of antibodies purchased from BioLegend, including anti-mouse CD3 (PerCP/Cyanine5.5, 17A2 clone), anti-mouse CD4 (Alexa Fluor 700, RM4-5 clone), anti-mouse CD19 (APC/Cyanine7, 6D5 clone), anti-mouse CD8a (Brilliant Violet 510™, 53-6.7 clone), anti-mouse CD107a (Brilliant Violet 421™, 1D4B clone) and the viability dye Near-IR. Following washing with PBS, the cells were fixed and permeabilized with Cytofix/Cytoperm (BD Biosciences, USA), washed with Perm/Wash buffer (BD Biosciences, USA), and stained with anti-mouse IFN-γ (PE, XMG1.2 clone), anti-mouse TNF-α (APC, MP6-XT22 clone), anti-mouse IL-2 (PE/Cyanine7, JES6-5H4 clone) and anti-mouse IL-4 (Brilliant Violet 605™, 11B11 clone). The cells were washed and resuspended in PBS before acquisition on a FACS Canto™ flow cytometer (BD Biosciences, USA).

### Evaluation of germinal center responses by flow cytometric analysis

The draining inguinal LNs were homogenized and filtered through a 70-μm cell strainer, and 2×10^6^ cells were stained with the viability dye Near-IR and blocked with anti-mouse CD16/32 (S17011E clone, BioLegend, USA). Subsequently, the cells were stained with anti-mouse CD19 (Brilliant Violet 605™, 6D5 clone), anti-mouse GL7 (PE, GL7 clone), anti-mouse Fas (APC, SA367H8 clone), anti-mouse CD4 (FITC, RM4-5 clone), anti-mouse PD-1 (Brilliant Violet 421™, 29F.1A12 clone), anti-mouse CXCR5 (PE/Cyanine7, L138D7 clone) and anti-mouse CD3 (PerCP/Cyanine5.5, 17A2 clone), which were all purchased from BioLegend. After staining, the cells were fixed, resuspended in PBS, and then acquired on a BD FACS Canto™ flow cytometer.

### Detection of viral loads in tissues by qRT–PCR

The viral loads in the spleen and liver were determined by qRT-PCR. In brief, viral RNA was extracted using a RNeasy Mini Kit (Qiagen, Germany) according to the manufacturer’s instructions. Reverse transcription was performed using PrimeScript™ RT Master Mix (Takara, Japan). qPCR was conducted using TaqMan™ Universal Master Mix II (Thermo Fisher Scientific, USA) with primers and probes described previously: forward primer 5’-GAAAATTCCTGAAACACATGG-3’, reverse primer 5’-ACTTCCTTGCATCATCTGATG-3’ and probe FAM- CAATGTAAGGGGCCTGTGTGGACTTGTG-BHQ1-3’ (35). The amount of viral RNA was normalized to a standard curve generated from a plasmid containing a partial cDNA sequence of the RVFV L gene.

### Histopathology and immunohistochemistry

Collected spleen and liver tissues were fixed with a 4% paraformaldehyde solution at room temperature for 48 h, embedded in paraffin, and sectioned. Each sample was examined in duplicate. One section was stained with hematoxylin and eosin (H&E) to observe microscopic lesions, and the other section was stained with a monoclonal antibody specific for the RVFV Gn protein to detect the RVFV Gn antigen. Images were captured using a Pannoramic 250 FLASH and were processed using SlideViewer software.

### *In vivo* luciferase activity

To detect the *in vivo* infection of Ad4, interferon-α/β receptor-deficient A129 mice (n = 5) were inoculated with Ad4 viral vector expressing the luciferase gene *via* intramuscular at a dose of 10^7^ IFU. At indicated time post inoculation, animals were injected intraperitoneally with luciferase substrate (Perkin Elmer). After reaction for 10 minutes, fluorescence signals were detected by IVIS Spectrum instrument and the fluorescence signals were quantified using Living Image 3.0.

### Statistical analysis

All statistical analyses were conducted using GraphPad Prism 8.0. Data are shown as the mean ± SEM. One-way ANOVA with Tukey’s multiple comparison test was performed for multiple-group (>2) comparisons, and a two-tailed t test was performed for two-group comparisons. *P* values <0.05 were considered significant (*P < 0.05, **P < 0.01, ***P < 0.001 and ****P < 0.0001; ns, no significance).

## Results

### Construction and identification of Ad4-GnGc

**Figure 1:**
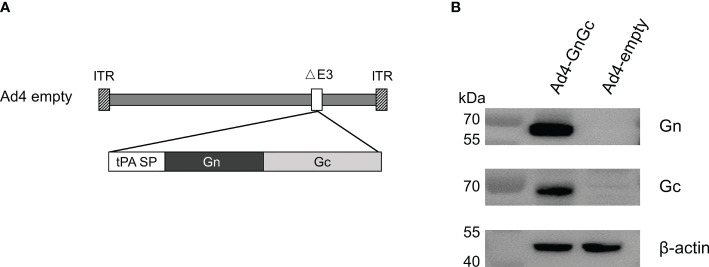
Construction and characterization of the RVFV vaccine Ad4-GnGc. **(A)** Schematic of the Ad4-GnGc vaccine expressing the RVFV glycoproteins Gn and Gc. **(B)** The expression of the Gn and Gc proteins in Vero E6 cells. Vero E6 cells were infected with Ad4-GnGc or Ad4-empty at a multiplicity of infection (MOI) of 10, and western blotting was performed 48 h after infection. ITR, inverted terminal repeat; tPA SP, tissue plasminogen activator signal peptide.

To develop a prophylactic vaccine against RVFV, an E3-deleted replication-competent Ad4 vector carrying the Gn and Gc glycoproteins of the RVFV MP-12 strain (accession number DQ380208.1) within its E3 region was constructed and named Ad4-GnGc (**Figure 1A**). To increase antigen expression, the Gn- and Gc-encoding sequences were codon optimized, and the viral origin signal was substituted with the tPA signal peptide. Ad4-GnGc was successfully rescued and propagated in HEK 293 cells. Infection of Vero E6 cells with Ad4-GnGc resulted in efficient expression of the antigenic proteins (**Figure 1B**). Besides, the variability of antigenic proteins expression between batches was also analyzed, two batches of Ad4-GnGc were randomly selected and qualitatively detected by western blot, and the results showed that the antigenic proteins can be efficiently expressed in both batches (**Supplementary Figure 1**).

### Ad4-GnGc elicits strong humoral and cellular immune responses in BALB/c mice

**Figure 2:**
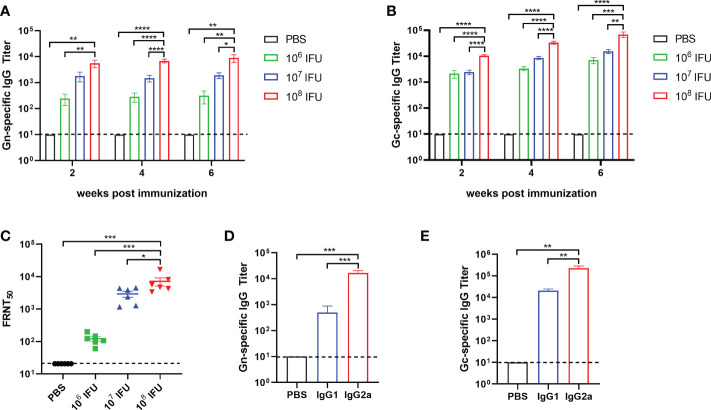
Humoral immune response in Ad4-GnGc-vaccinated mice. Groups of BALB/c mice (n = 6) were immunized with a single dose of 10^6^, 10^7^ or 10^8^ IFU of Ad4-GnGc or with PBS *via* the intramuscular route. Sera were collected 2, 4, and 6 weeks after immunization. RVFV Gn- **(A)** and Gc-**(B)** specific IgG antibody titers were determined by ELISA. **(C-E)** Sera from the high dose group collected on day 28 after immunization were used to determine the titers of neutralizing antibodies **(C)** and Gn- **(D)** and Gc-specific **(E)** IgG subclasses. Data are shown as the mean ± SEM. *P* values were calculated by one-way ANOVA with multiple comparison tests. *P < 0.05, **P < 0.01, ***P < 0.001, ****P < 0.0001.

**Figure 3:**
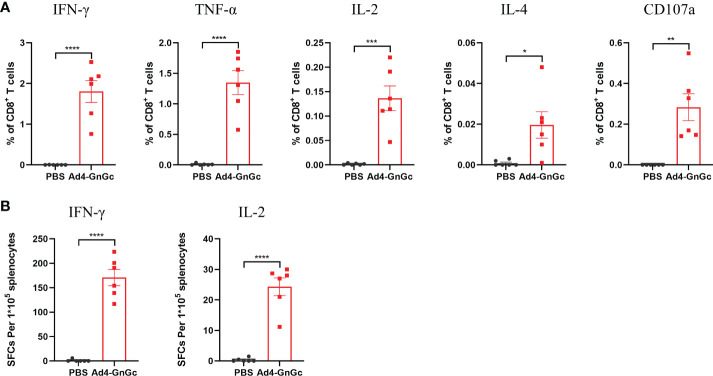
RVFV-specific T-cell immune response in Ad4-GnGc-vaccinated mice. BALB/c mice (n = 6) were immunized intramuscularly with a single dose of 10^8^ IFU of Ad4-GnGc or PBS. Two weeks after vaccination, the mice were sacrificed, and splenocytes were collected. **(A)** The percentages of CD8^+^ T cells secreting IFN-γ, TNF-α, IL-2, IL-4 or CD107a were determined by ICCS. **(B)** An ELISPOT assay was performed to assess IFN-γ and IL-2 secretion by mouse splenocytes. Data are shown as the mean ± SEM. *P* values were analyzed with an unpaired Student’s *t* test. *P < 0.05, **P < 0.01, ***P < 0.001, ****P < 0.0001.

To assess the immunogenicity of Ad4-GnGc, six- to eight-week-old female BALB/c mice were immunized intramuscularly with a single dose of 10^6^, 10^7^ or 10^8^ IFU of Ad4-GnGc or PBS. After immunization, mice were randomly selected for temperature and weight monitoring at regular intervals, and no abnormality was found. Serum samples were collected 2, 4, 6 and 18 weeks after immunization and subjected to antibody detection. As a result, virus-specific IgG was induced by Ad4-GnGc in a dose-dependent manner, and even the low dose could generate strong IgG responses. The serum Gc-specific IgG titer was generally higher than that of Gn in each group at the different timepoints (**Figures 2A, B** **)**. Besides, we also found that Gn- and Gc-specific IgG antibody titers could remain at high levels until 18 weeks after immunization, which indicated that Ad4-GnGc could elicit a durable antibody response (**Supplementary Figure 2**). The anti-RVFV NAb titers induced by the vaccine were determined with the rescued reporter virus rMP-12-eGFP by a 50% focus reduction neutralization test (FRNT_50_). As expected, the NAb titers were also induced in a dose-dependent manner, consistent with the above IgG responses (**Figure 2C**). Previous studies have shown that IgG1 is associated with Th2 responses, while IgG2a often indicates Th1 responses in mice (36, 37). To determine which type of immune response was induced by Ad4-GnGc, Gn- and Gc-specific IgG1 and IgG2a were detected at 4 weeks post-vaccination in the high-dose group. The results indicated that the vaccine could induce not only IgG1 (Th2) responses but also IgG2a (Th1) responses, with the IgG2a titers being significantly higher than the IgG1 titers, which implied a Th1-biased response (**Figures 2D, E** **)**. To further evaluate the cellular immune response elicited by Ad4-GnGc, splenocytes from high dose-immunized mice were collected two weeks after vaccination and tested with ICCS and ELISPOT assays. The vaccinated mice could produce both Th1 and Th2 responses, and the proportions of CD8^+^ T cells that secreted IFN-γ, TNF-α, IL-2 or IL-4 were all significantly increased. However, it’s worth mentioning that the expression level of IL-4 is significantly lower than that of IFN-γ, TNF-α and IL-2, indicating a Th1-biased response, which was in accordance with the results of other recombinant adenovirus-vectored vaccines (38, 39). Besides, CD8^+^ T cells secreting CD107a, which has been described as a marker of cytotoxic CD8^+^ T-cell degranulation and cytotoxic activity (40), were also significantly induced (**Figures 3A, B** and **Supplementary Figure 3**). However, the corresponding CD4^+^ T cell populations were barely detectable (data not shown), which was in accordance with previous results (16, 41). Considering the background of Ad4-empty vector, the ability of Ad4-empty vector to induce an immune response was also evaluated, and our results showed that the background of antibody and cellular responses in Ad4-empty vector control group was consistent with the results of the PBS grou**p (Supplementary Figure 4**).

**Figure 4:**
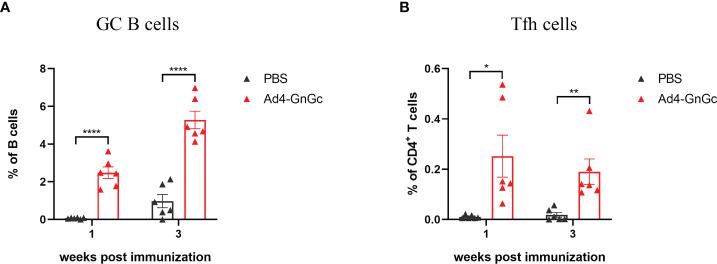
Ad4-GnGc vaccine elicits strong GC B cell and Tfh cell responses. BALB/c mice (n = 12) were immunized with a single dose of 10^8^ IFU of Ad4-GnGc or PBS *via* intramuscular route. Half of the mice in each group were sacrificed 1 week after immunization, and the other half were sacrificed 3 weeks after immunization. The inguinal lymph nodes were collected. **(A)** The proportion of GC B cells, defined as live CD3^-^CD19^+^GL7^+^Fas^+^ cells, was determined by flow cytometry. **(B)** The frequency of Tfh cells, defined as live CD3^+^CD4^+^PD-1^+^CXCR5^+^ cells, was measured in each group. Data are displayed as the mean ± SEM. *P* values were analyzed with an unpaired Student’s *t* test. *P < 0.05, **P < 0.01, ****P < 0.0001.

Germinal centers (GCs) are important microanatomical sites of B-cell mutation and antibody affinity maturation induced during an immune response and are tightly regulated by T follicular helper (Tfh) cells, which enable the survival, proliferation, and differentiation of GC B cells through delivery of costimulatory molecules and cytokines (42, 43). The effective generation of Tfh cells can result from improved antigen presentation to CD4^+^ T cells. We hypothesized that the robust immune responses induced by Ad4-GnGc might be attributed to superior GC formation in the secondary lymphoid organs. To test our hypothesis, BALB/c mice were immunized intramuscularly with a single dose of 10^8^ IFU of Ad4-GnGc, and GC B cells and Tfh cells were evaluated in the inguinal lymph nodes (LNs) by flow cytometry. We found a significantly increased percentage of total GC B cells in the vaccine-immunized group compared with the PBS group at 7 days post-immunization, and the response remained at a high level until day 21 (**Figure 4A** and **Supplementary Figure 5**). Additionally, a higher percentage of Tfh cells was detected in the inguinal LNs on days 7 and 21 (**Figure 4B** and **Supplementary Figure 5**). Together, these data suggest that Ad4-GnGc induces strong GC responses, which can last for at least three weeks after immunization.

### Pre-existing immunity to the Ad5 vector has no effect on the efficacy of Ad4-GnGc

**Figure 5:**
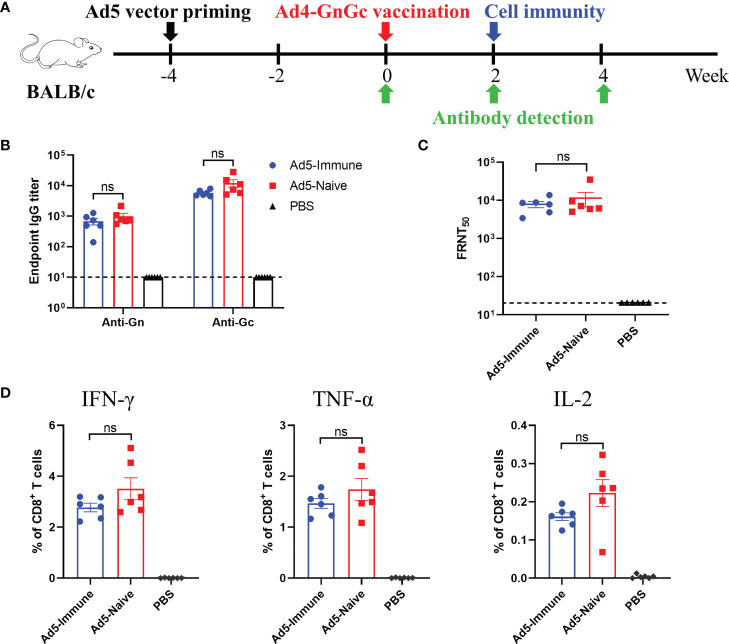
Pre-existing immunity to Ad5 has no effect on the efficacy of Ad4-GnGc. BALB/c mice (n = 12) were primed with 10^8^ IFU of Ad5 vector at week -4 by intramuscular injection to induce anti-vector immune responses. Naive animals were maintained as vector-naive controls. On week 0, animals were vaccinated intramuscularly with 10^8^ IFU of Ad4-GnGc, and PBS-treated mice were maintained as a negative control. In each group, six mice were used for serum collection at different time after immunization for binding antibody or neutralizing antibody detection, and the other six mice were used for cytokine detection. **(A)** Schematic of the experimental design. **(B)** Serum collected on day 14 after Ad4-GnGc immunization was used to measure RVFV Gn- and Gc-specific IgG antibody titers. **(C)** Four weeks after vaccination, serum neutralizing antibody titers were determined using infectious RVFV by the FRNT. **(D)** Two weeks after Ad4-GnGc vaccination, six mice from each group were sacrificed and the splenocytes were collected to determine the percentages of IFN-γ-, TNF-α- and IL-2-secreting CD8^+^ T cells by ICCS. Data are shown as the mean ± SEM. Significance was calculated using an unpaired Student’s *t* test. n.s., not significant.

Pre-existing immunity to Ad5 is a major concern in the application of Ad5-vectored vaccines because it could weaken the cellular and humoral immune responses induced by recombinant vaccines (23, 44). To investigate whether pre-existing immunity to the Ad5 vector could interfere with the efficacy of Ad4-GnGc, we inoculated BALB/c mice with 10^8^ IFU of Ad5 on week -4 to induce an anti-vector immune response (**Figure 5A**). Four weeks after the inoculation (week 0), Ad5-specific IgG was detected by ELISA, and the results showed that all the Ad5-immunized mice had very high levels of anti-vector antibodies compared to uninoculated animals (**Supplementary Figure 6**). On week 0, the mice in the Ad5-immune and Ad5-naive groups were vaccinated with 10^8^ IFU of Ad4-GnGc. As a negative control, the mice in the PBS group received an equal volume of PBS (**Figure 5A**). Our results showed that Ad4-GnGc induced similar levels of Gn- and Gc-specific IgG antibodies as well as NAbs specific for RVFV in both the Ad5-immune and Ad5-naive groups (**Figures 5B, C** **)**. In addition, a Th1 cytokine profile of IFN-γ-, TNF-α- and IL-2-secreting CD8^+^ T cells was also induced, with no significant difference between the Ad5-immune and Ad5-naive groups (**Figure 5D**). Taken together, the above results demonstrated that pre-existing immunity to the Ad5 vector did not dampen the immunogenicity of Ad4-GnGc. Therefore, Ad4-GnGc is similarly effective in inducing humoral and cellular immune responses in both Ad5-seropositive subjects and Ad5-seronegative subjects.

### Ad4-GnGc vaccination can provide protection against RVFV challenge in A129 mice

**Figure 6:**
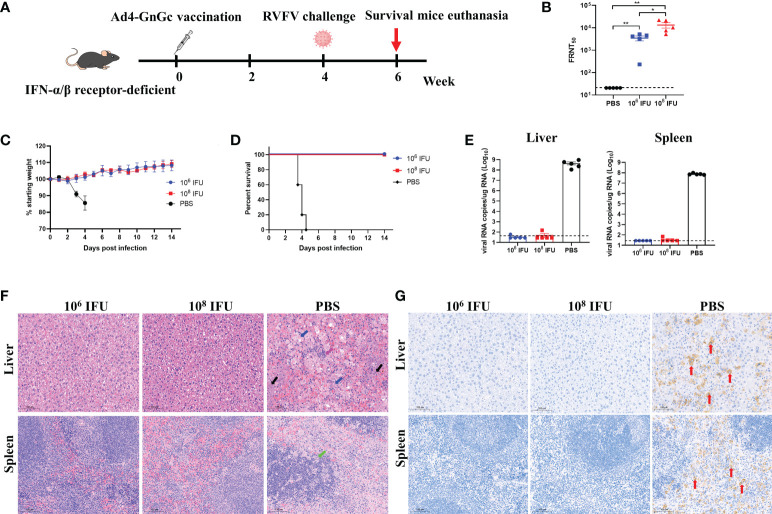
Protective effect of Ad4-GnGc against RVFV challenge in IFN receptor-deficient mice. Mice (n = 5) received one dose of 10^6^ or 10^8^ IFU of Ad4-GnGc or PBS *via* the intramuscular route. Four weeks after vaccination, the mice were challenged intraperitoneally with 2×10^4^ TCID_50_ of RVFV rMP-12 strain. The livers and spleens from PBS group (at the time of death) and vaccine-immunized groups (14 days after challenge) were collected and analyzed with viral RNA load, histopathological and immunohistochemical assays. **(A)** Schematic diagram of the immunization and challenge schedule. **(B)** Four weeks after vaccination (before virus challenge), serum neutralizing antibody titers were determined using infectious RVFV. **(C)** Mouse body weight change after RVFV challenge. **(D)** Mortality and survival curves of mice. **(E)** The viral loads in the liver and spleen following virus challenge were determined by qRT–PCR. **(F)** Representative histopathology (H&E) of livers and spleens from RVFV-infected mice. Hepatocyte steatosis is indicated by blue arrows; inflammatory cell infiltration is indicated by purple arrows; hepatocyte nuclear vacuolation and apoptosis are indicated by black arrows; splenic nodule atrophy is indicated by green arrows. Scale bar, 100 μm. **(G)** Representative immunohistochemical (IHC) staining of liver and spleen tissues with RVFV Gn-specific monoclonal antibodies. Red arrows indicate RVFV-positive signals. Scale bar, 100 μm. Data are shown as the mean ± SEM. *P* values were calculated by one-way ANOVA with multiple comparison tests. *P < 0.05, **P < 0.01.

Interferon-α/β receptor-deficient A129 mice have been shown to be highly susceptible to many viruses and are frequently used for the evaluation of antivirals and vaccines for virus infections, such as infection with Zika virus or RVFV (30, 32, 45). Considering the species restriction of adenovirus, we first explored the susceptibility of this animal model to Ad4 by bioluminescence imaging analysis. A129 mice were inoculated intramuscularly with Ad4 viral vector expressing the luciferase gene at a dose of 10^7^ IFU, then the expression of luciferase was detected at different time points after inoculation, and our results showed that the luciferase can be expressed effectively *in vivo* and peaks at 1-2 days after inoculation, which implies that A129 mouse is a permissive host for Ad4 construct (**Supplementary Figure 7**). To further evaluate the *in vivo* protective efficacy of Ad4-GnGc, A129 mice that received a single dose of 10^6^ IFU, 10^8^ IFU or PBS were challenged intraperitoneally with 2×10^4^ median TCID_50_ of RVFV rMP12 strain 4 weeks after vaccination (**Figure 6A**). The A129 mice in both the low- and high-dose groups generated high titers of NAbs four weeks after immunization (pre-challenge), as determined by live virus neutralization assays (**Figure 6B**). The body weight of each mouse was recorded daily after RVFV challenge, and the results showed that the body weights of both the low- and high-dose groups increased steadily, while that of the PBS group decreased significantly, with 9%-19% body weight loss (**Figure 6C**). All animals in the PBS group displayed clinical signs, including ruffled fur, lethargy, and eye discharge, and died within 3-5 days of the challenge. Among them, two of the mice died before reaching the humane endpoint (3.5 and 4 days after challenge, respectively), the other three were euthanized when clinical scores reached humane endpoint (3.5, 4 and 4.5 days after challenge, respectively) (**Supplementary Table 1**). The viral loads in the liver and spleen could reach 10^8.66^ and 10^7.88^ copy equivalents per microgram of total RNA respectively at the time of death (**Figures 6D, E** **)**. Whereas mice in the vaccine-immunized groups remained healthy with no clinical symptoms and survived throughout the experiment, that is 14 days after challenge. Then these mice were euthanized, viral loads in the liver and spleen were also measured, and we found that almost no viral RNA could be detected, indicating that immunization of A129 mice with Ad4-GnGc confers protection against RVFV challenge (**Figures 6D, E** **)**. More importantly, the mice inoculated with PBS developed severe microscopic liver lesions at the time of death, characterized by scattered necrotic foci, hepatocyte nuclear vacuolation and apoptosis, hepatocyte steatosis and inflammatory cell infiltration. Meanwhile, scattered atrophy of splenic nodules could be observed in spleen tissues. However, no such pathological changes were observed in liver or spleen sections from all Ad4-GnGc-immunized animals at the time of euthanasia (**Figure 6F**). Correspondingly, immunohistochemical assay results showed that many RVFV-positive signals were detected in PBS-inoculated mice, while no virus was observed in the liver or spleen of Ad4-GnGc-vaccinated mice (**Figure 6G**). These data demonstrated that a single low-dose vaccination with Ad4-GnGc could efficiently protect mice from lethal infection.

## Discussion

Preventing RVFV epidemic outbreaks requires prophylactic vaccines that can rapidly induce protective host immune responses, including humoral and cellular immune responses. The glycoproteins Gn and Gc of RVFV, which mediate viral entry and contain the major epitopes recognized by NAbs (46), have been considered the principal antigen targets for RVFV vaccines (12). Based on these observations, many vaccine candidates, including DNA vaccines (47), recombinant viral vectors such as recombinant adenoviral vectors (17, 48) and recombinant modified vaccinia virus Ankara (MVA) (32, 41), have shown different degrees of immunogenicity and protective efficacy in different animal models.

Adenovirus-vectored vaccines have many advantages and are widely used in vaccine development (49, 50). We and others have previously evaluated the safety and immunogenicity of adenovirus-vectored vaccines for Ebola virus (21), respiratory syncytial virus (51), and SARS-CoV-2 (23) in clinical trials. To date, two recombinant adenovirus-vectored RVFV vaccines have been reported: the Ad5 vector-based vaccine CAdVax-RVF (48) and the chimpanzee adenovirus vector-based vaccine ChAdOx1-GnGc (16, 52). However, several limitations have been found for each vaccine. The high prevalence of pre-existing immunity to the Ad5 vector in humans may limit Ad5 vector-based RVFV vaccine performance. Although ChAdOx1-GnGc is not affected by extensive pre-existing anti-vector immunity, its immune effect was found to be significantly lower than that of CAdVax-RVF (16). Compared with nonreplicating vector vaccines, replicating vector vaccines have many advantages because they may combine the safety of inactivated or subunit vaccines with the efficacy of live attenuated vaccines (53). In this study, we bioengineered Ad4, which has already been used in human disease prevention and has good safety records in humans (24, 26, 54), as a replication-competent vector to generate an RVFV vaccine, Ad4-GnGc, and demonstrated that a single immunization with the candidate vaccine could induce robust antibody and cell-mediated immune responses against RVFV in mice. As with most other adenovirus-vectored vaccines, the antibody response elicited by Ad4-GnGc was also durable (45, 55), with the binding antibodies remaining at high levels for more than four months. Besides, previous studies have shown that replication-competent adenovirus type four recombinant virus expressing influenza H5 hemagglutinin could induce prolonged evolution of the memory B cell response (27). Based on these, we speculated that as a replication-competent adenovirus-vectored vaccine, Ad4-GnGc could also induce memory B cell responses. Since vaccines are often administered during an RVF outbreak, the ability to provide two doses of any vaccine may be limited. Therefore, our results suggest that Ad4-GnGc may serve as a potential candidate vaccine for human clinical trials and susceptible animal species.

The prevalence of Ad5 in humans caused by natural exposure or prior vaccination may reduce the efficacy of Ad5-vectored vaccines. The positive rate of Ad5-specific NAbs in populations in America, Southern China, and Africa has been reported to be very high (56). To make matters worse, with the application of an Ad5-vectored coronavirus disease 2019 (COVID-19) vaccine, the rate might rise further. Although increasing the dose may overcome pre-existing Ad5 immunity (44) and induce sufficient antigen-specific immune responses, the risk of side effects might also increase. In our study, we inoculated BALB/c mice with 10^8^ IFU of Ad5 vector, which was significantly higher than dose experienced in natural infection. Four weeks after inoculation, when the Ad5 vector had induced a strong immune response, the mice were immunized with Ad4-GnGc, and our results showed that anti-vector immunity had no effect on the humoral or cellular immune responses induced by the vaccine. That is, under normal circumstances, the vaccine effectively circumvented the influence of pre-existing Ad5 immunity. These results suggested that Ad4-GnGc can be considered an alternative RVFV vaccine for use in Ad5 seropositive subjects or as a sequential booster vaccine after the subjects have been immunized with a recombinant Ad5-based vaccine.

Previous studies have shown that attenuated RVFV strains are highly virulent in interferon-α/β receptor-deficient mice (57), and this animal model has been widely used in RVFV vaccine evaluations (30, 32, 41). In the present research, we evaluated the protective immune response induced by the vaccine in this animal model. Although the detection time points of viral loads in the tissues were different, the results also showed that mice in PBS group died of RVFV infection, while virus in Ad4-GnGc-immunized mice was completely cleared, indicating that even low-dose (10^6^ IFU) Ad4-GnGc vaccination could protect mice against a lethal RVFV challenge. However, it needs to be noted that due to the lack of a wild-type virus, rMP-12 was rescued by a reverse genetic system according to previous research, and the growth kinetics of rMP-12 were similar to those of its parental virus MP-12, as shown by previous research (28). Thus, rMP-12 was used instead of wild-type MP-12 in the animal challenge experiment. In addition, the reporter virus rMP-12-eGFP was also rescued and used in the microneutralization assay; although it had a slightly lower titer, its growth kinetics were similar to those of rMP-12 (data not shown), which is also consistent with previous research (28).

Our study demonstrated that the candidate vaccine Ad4-GnGc could induce strong immune responses in mice. However, it’s worth mentioning that in order to facilitate experimental operation and feeding management, all animals used in this study are female, whether Ad4-GnGc has similar immunogenicity in male mice deserves further investigation. Besides, since RVFV can infect a variety of animals, especially ruminants, the ability of the candidate vaccine to induce immune responses and its protective properties in different animal models, such as sheep, cattle, and primates, need to be further evaluated. Another limitation of our study was that the challenge experiments were based on the attenuated RVFV strain MP-12, and further challenge experiments with a virulent strain will make the data on the protective efficacy more comprehensive. Overall, the Ad4-based replication-competent vaccine displayed excellent immunogenicity and protective efficacy in mice, and pre-existing Ad5 immunity had no effect on its efficacy, indicating that Ad4-GnGc is a very promising candidate vaccine against RVFV. Further work is needed to confirm these results in a larger study and to elucidate the initiation and duration of immunity, as well as identify any variations in vaccine efficacy among different animal models.

## Data availability statement

The original contributions presented in the study are included in the article/**Supplementary Material**. Further inquiries can be directed to the corresponding authors.

## Ethics statement

The animal study was reviewed and approved by the Institutional Experimental Animal Welfare and Ethics Committee of Sino Animal (BeiJing) Science and Technology Development Co. Ltd.

## Author contributions

CY, JL, and WC designed, directed and supervised the entire study. TB performed most of the experiments, analyzed all results, and wrote the manuscript. BW constructed the Ad4 vector system. GF, MH, and YC performed the immunization and protection experiment. TF and SL assisted the mouse immunization and evaluation experiments. All authors contributed to the article and approved the submitted version.

## Funding

This work was supported by the National Natural Science Foundation of China (82101919) and Beijing Municipal Science and Technology Project (Z201100005420024).

## Conflict of interest

The authors declare that the research was conducted in the absence of any commercial or financial relationships that could be construed as a potential conflict of interest.

## Publisher’s note

All claims expressed in this article are solely those of the authors and do not necessarily represent those of their affiliated organizations, or those of the publisher, the editors and the reviewers. Any product that may be evaluated in this article, or claim that may be made by its manufacturer, is not guaranteed or endorsed by the publisher.
