# Supplementary material for: Single-dose of a replication-competent adenovirus-vectored vaccine provides sterilizing protection against Rift Valley fever virus challenge
Source: Front Immunol. 2022 Nov 11;13:907675. doi: 10.3389/fimmu.2022.907675 (PMC9691644; doi:10.3389/fimmu.2022.907675)
Supplement: Supplementary file 1 [file DataSheet_1.docx]

Supplementary Material

## Supplementary Figures


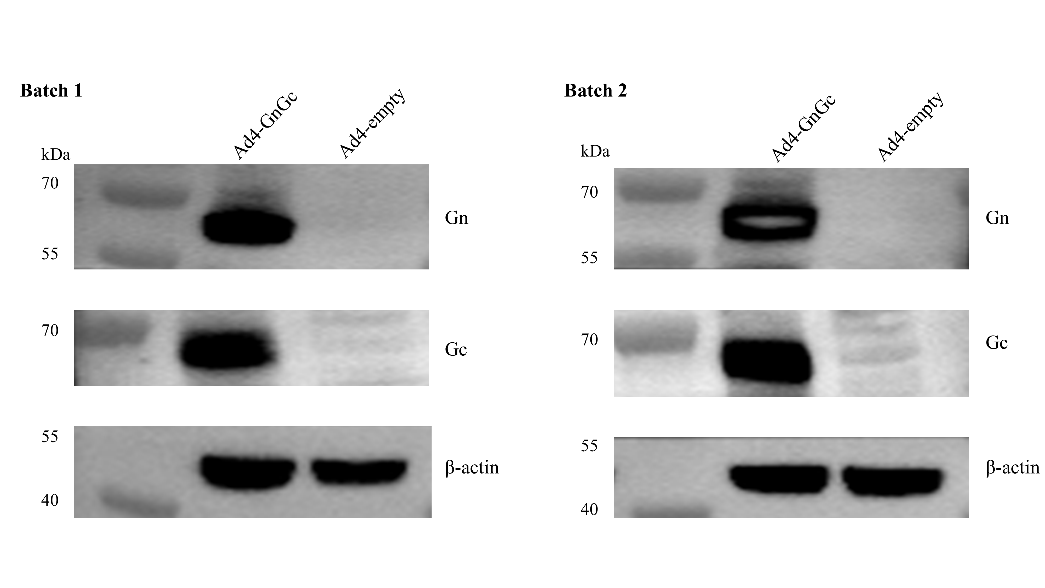


**Supplementary Figure 1**. The variability of Gn and Gc expression between two batches. Vero E6 cells were infected with two randomly selected batches of Ad4-GnGc or Ad4-empty at a multiplicity of infection (MOI) of 10, and western blotting was performed 48 h after infection.


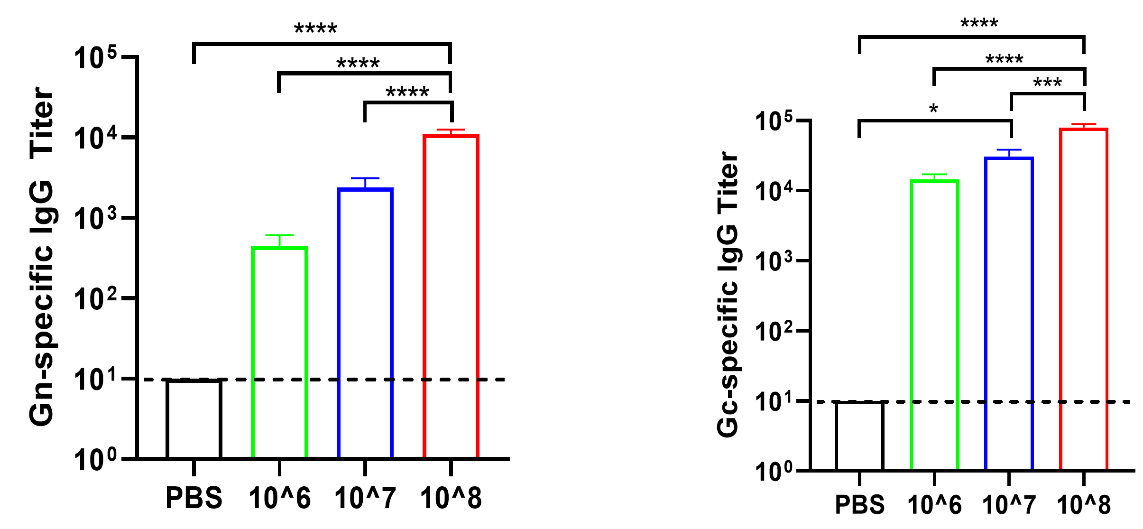


**Supplementary Figure 2**. RVFV Gn- (left) and Gc- (right) specific IgG antibody titers 18 weeks after immunization. Groups of BALB/c mice (n = 6) were immunized with a single dose of 10^6^, 10^7^ or 10^8^ IFU of Ad4-GnGc or with PBS via the intramuscular route. Serum samples collected 18 weeks after immunization were used for binding antibody detection by ELISA. Data are shown as the mean ± SEM. *P* values were calculated by one-way ANOVA with multiple comparison tests. **P* < 0.05, ****P* < 0.001, *****P* < 0.0001.


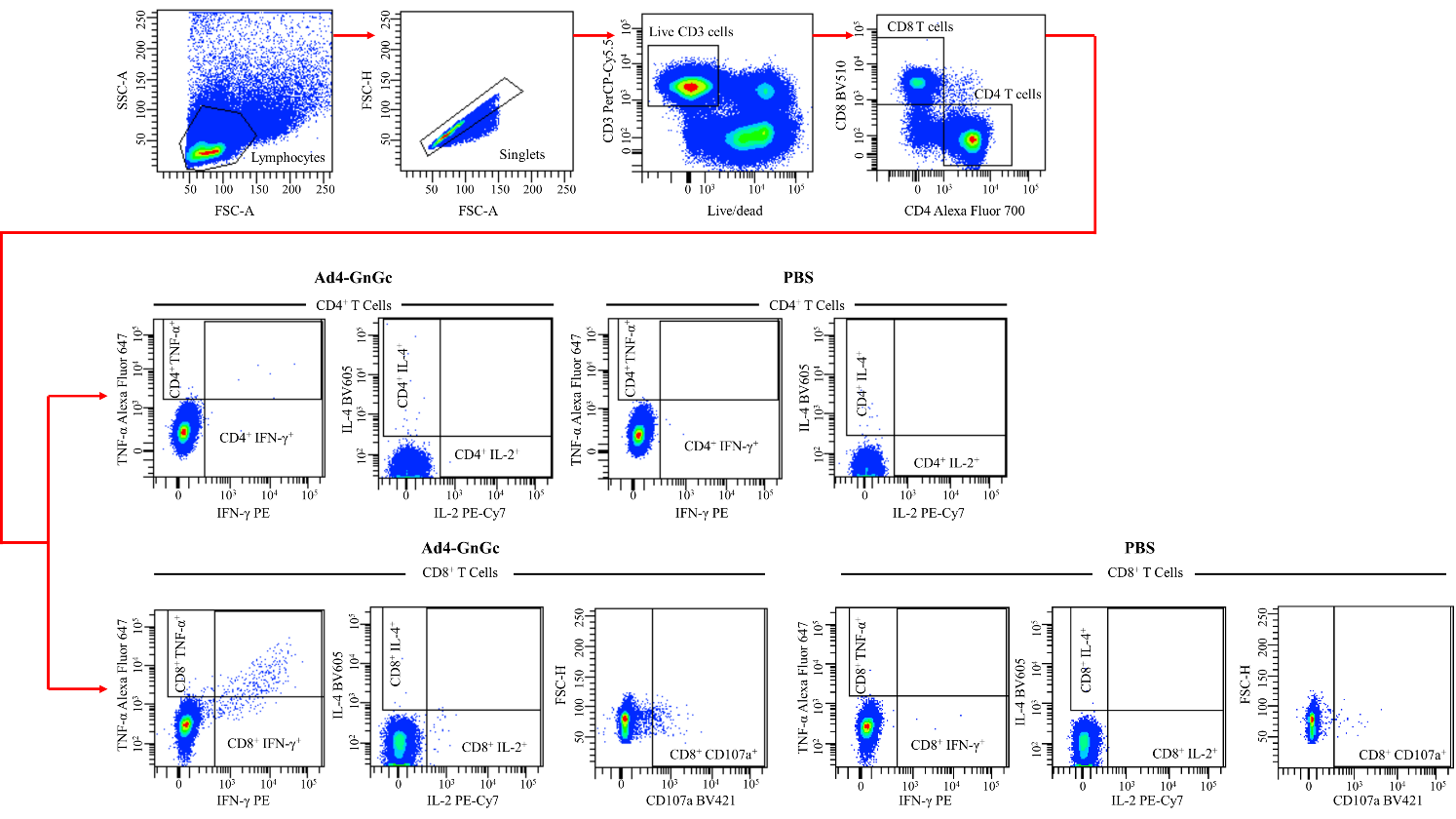


**Supplementary Figure 3.** Representative gating strategy for intracellular cytokine staining. BALB/c mice (n = 6) were immunized intramuscularly with a single dose of 10^8^ IFU of Ad4-GnGc or PBS. Two weeks after vaccination, the mice were sacrificed. Then the splenocytes were collected and stimulated with RVFV Gn and Gc peptides. After staining with different fluorochrome-conjugated antibodies, flow cytometric analysis was performed to assess multifunctional cytokine-positive T cell subsets.

**
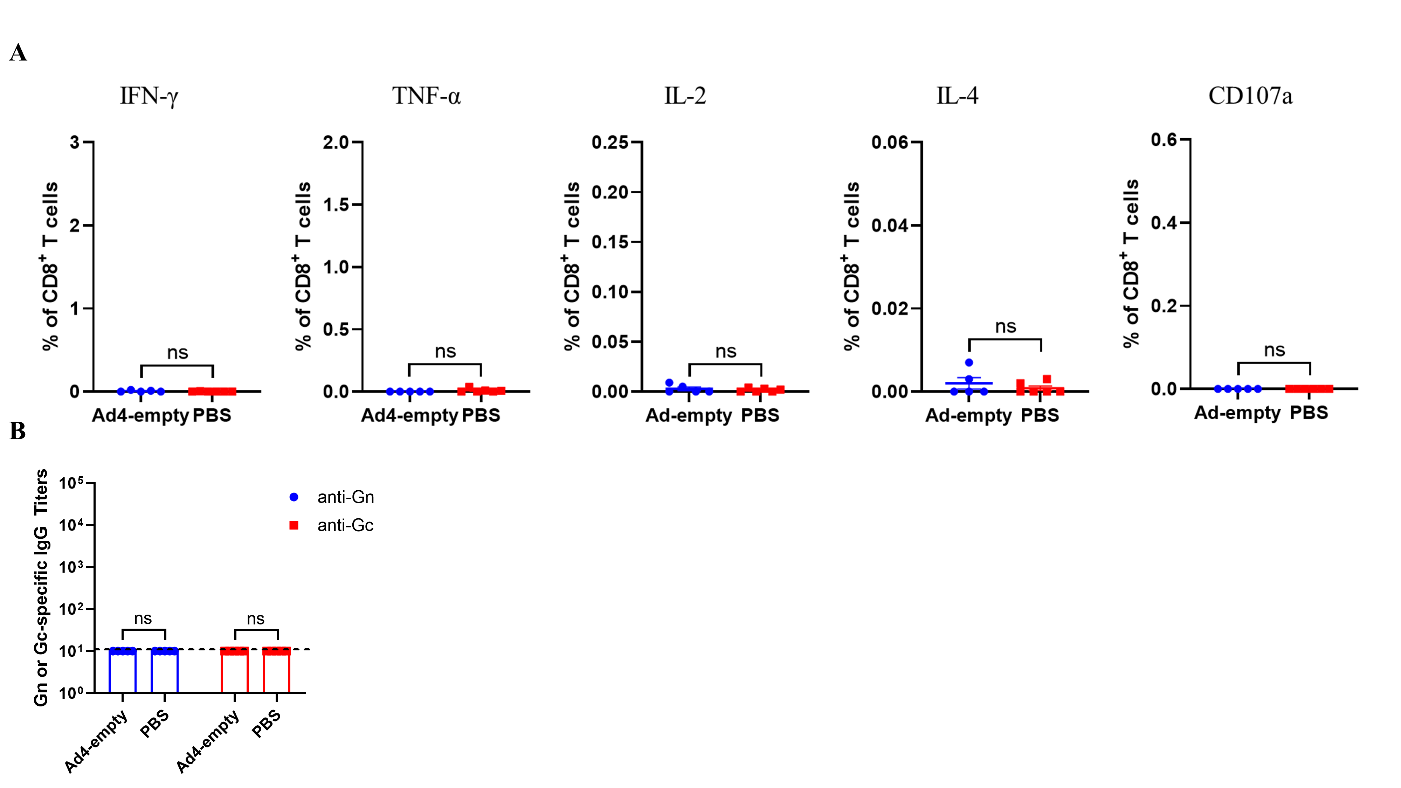
**

**Supplementary Figure 4.** Cellular and humoral immune responses in Ad4-empty vector-inoculated mice. BALB/c mice (n = 6) were immunized with a single dose of 10^8^ IFU of Ad4-empty vector or PBS via the intramuscular route. Two weeks after vaccination, the mice were sacrificed, and splenocytes were collected. **(A)** The percentages of CD8^+^ T cells secreting IFN-γ, TNF-α, IL-2, IL-4 or CD107a were determined by ICCS. **(B)** Sera collected 4 weeks after immunization were used to determine Gn- and Gc-specific IgG antibody titers. Data are shown as the mean ± SEM. *P* values were analyzed with an unpaired Student’s *t* test.


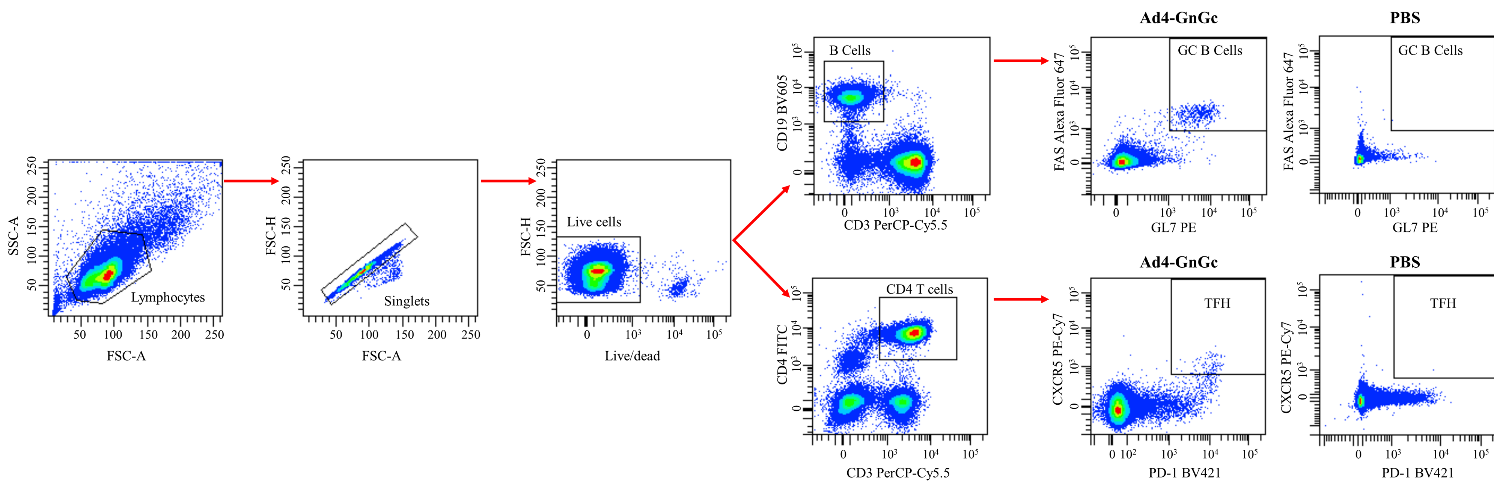


Supplementary Figure 5. Representative gating strategy for GC B cells and Tfh cells. BALB/c mice (n = 12) were immunized with a single dose of 10^8^ IFU of Ad4-GnGc or PBS via the intramuscular route. Six mice in each group were sacrificed 1 week after immunization, and the remaining mice were sacrificed 3 weeks after immunization. The inguinal lymph nodes were collected. Lymphocytes from the inguinal lymph nodes were stained and analyzed by flow cytometry. GC B cells were defined as live CD3^-^CD19^+^GL7^+^Fas^+^ cells, and Tfh cells were defined as live CD3^+^CD4^+^PD-1^+^CXCR5^+^ cells.


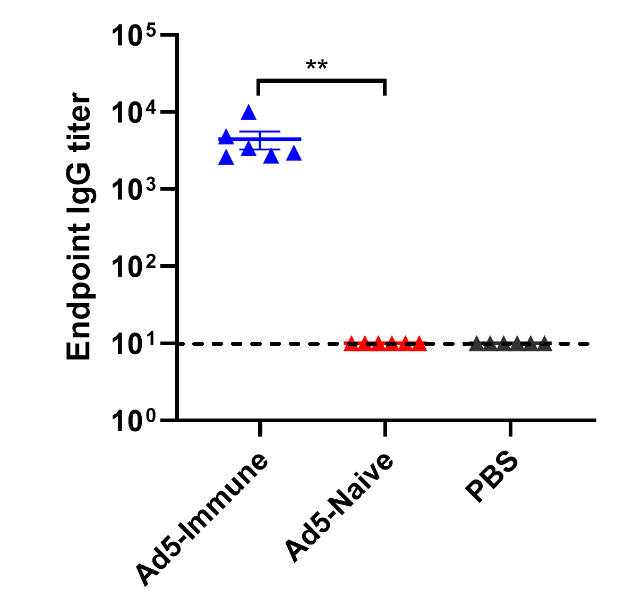


Supplementary Figure 6. Ad5 hexon-specific IgG antibody titers. Groups of BALB**/**c mice were intramuscularly inoculated with 10^8^ IFU of Ad5 vector, and sera were collected four weeks after inoculation. Ad5 hexon-specific IgG antibody titers were determined by ELISA. Data are shown as the mean ± SEM. *P* values were analyzed with an unpaired Student’s *t* test. ***P* < 0.01.


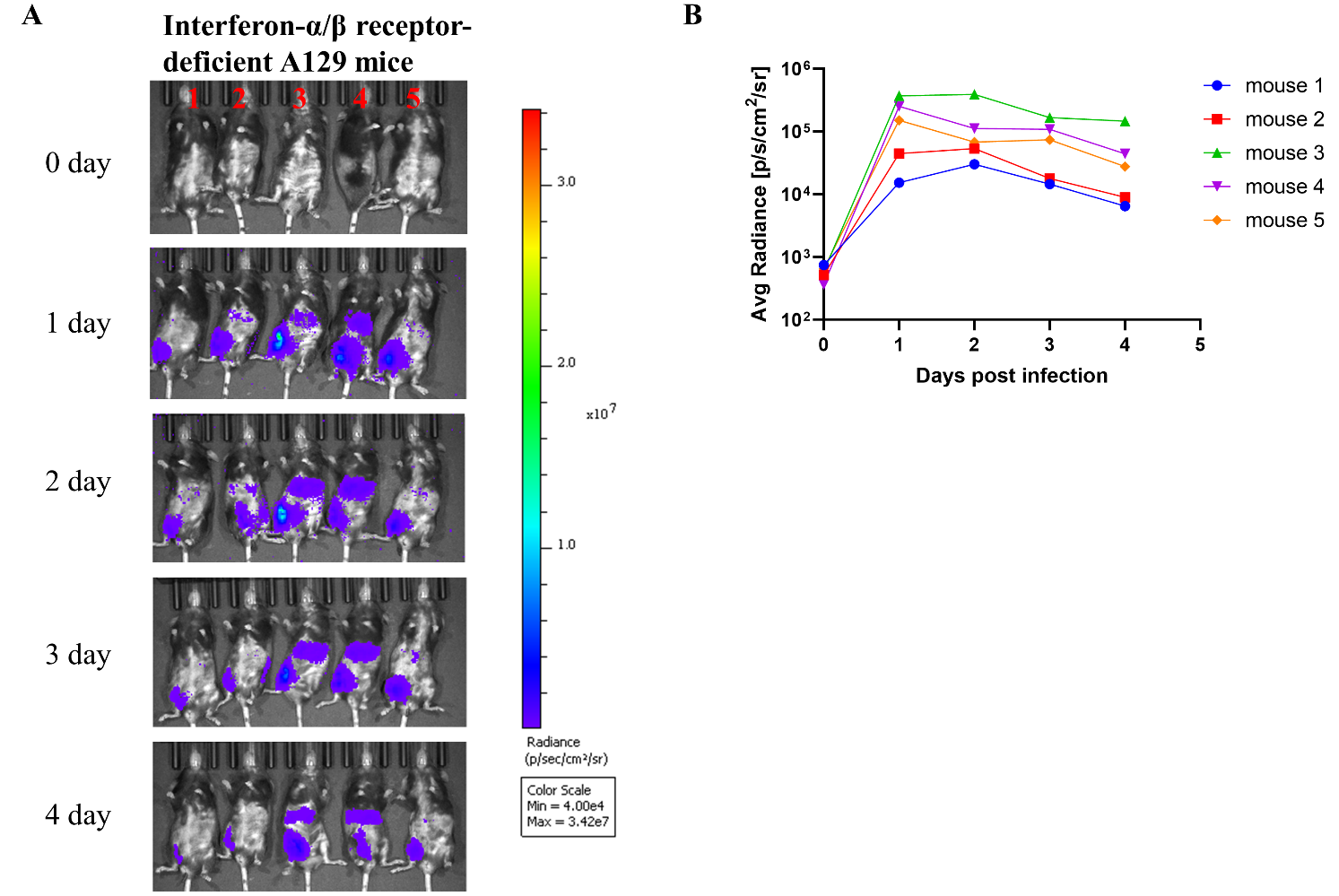


Supplementary Figure 7. *In vivo* infection of Ad4. Ad4 viral vector expressing the luciferase gene were used to infect interferon-α/β receptor-deficient A129 mice at a dose of 10^7^ IFU. (A) The expression of luciferase was detected at different time points after infection. The relative of bioluminescence was shown in pseudocolor, with red and purple representing the strongest and weakest photo fluxes, respectively. (B) Values of average radiance for five mice at different time points after inoculation.

**Supplementary Table 1∣**Clinical scores of mice in PBS group before death or euthanasia.

**Day 0 post infection**

| **Clinical symptoms** | | **Mouse 1** | **Mouse 2** | **Mouse 3** | **Mouse 4** | **Mouse 5** |
| --- | --- | --- | --- | --- | --- | --- |
| rough hair | Absent (0) | √ | √ | √ | √ | √ |
|  | Slightly (1) |  |  |  |  |  |
|  | Markedly (2) |  |  |  |  |  |
| activity | Normal (0) | √ | √ | √ | √ | √ |
|  | Slightly reduced (1) |  |  |  |  |  |
|  | Reduced (2) |  |  |  |  |  |
|  | Severely reduced (3) |  |  |  |  |  |
| eye discharge | Absent (0) | √ | √ | √ | √ | √ |
|  | Slightly (1) |  |  |  |  |  |
|  | Moderate (2) |  |  |  |  |  |
|  | Severe (3) |  |  |  |  |  |
| body weight | No reduce (0) | √ | √ | √ | √ | √ |
|  | Slightly reduced (1) |  |  |  |  |  |
|  | Reduced (2) |  |  |  |  |  |
|  | Severely reduced (3) |  |  |  |  |  |
| Total score |  | 0 | 0 | 0 | 0 | 0 |

**Day 1 post infection**

| **Clinical symptoms** | | **Mouse 1** | **Mouse 2** | **Mouse 3** | **Mouse 4** | **Mouse 5** |
| --- | --- | --- | --- | --- | --- | --- |
| rough hair | Absent (0) | √ | √ | √ | √ | √ |
|  | Slightly (1) |  |  |  |  |  |
|  | Markedly (2) |  |  |  |  |  |
| activity | Normal (0) | √ | √ | √ | √ | √ |
|  | Slightly reduced (1) |  |  |  |  |  |
|  | Reduced (2) |  |  |  |  |  |
|  | Severely reduced (3) |  |  |  |  |  |
| eye discharge | Absent (0) | √ | √ | √ | √ | √ |
|  | Slightly (1) |  |  |  |  |  |
|  | Moderate (2) |  |  |  |  |  |
|  | Severe (3) |  |  |  |  |  |
| body weight | No reduce (0) | √ | √ | √ | √ | √ |
|  | Slightly reduced (1) |  |  |  |  |  |
|  | Reduced (2) |  |  |  |  |  |
|  | Severely reduced (3) |  |  |  |  |  |
| Total score |  | 0 | 0 | 0 | 0 | 0 |

**Day 2 post infection**

| **Clinical symptoms** | | **Mouse 1** | **Mouse 2** | **Mouse 3** | **Mouse 4** | **Mouse 5** |
| --- | --- | --- | --- | --- | --- | --- |
| rough hair | Absent (0) | √ | √ | √ | √ | √ |
|  | Slightly (1) |  |  |  |  |  |
|  | Markedly (2) |  |  |  |  |  |
| activity | Normal (0) | √ | √ | √ | √ | √ |
|  | Slightly reduced (1) |  |  |  |  |  |
|  | Reduced (2) |  |  |  |  |  |
|  | Severely reduced (3) |  |  |  |  |  |
| eye discharge | Absent (0) | √ | √ | √ | √ | √ |
|  | Slightly (1) |  |  |  |  |  |
|  | Moderate (2) |  |  |  |  |  |
|  | Severe (3) |  |  |  |  |  |
| body weight | No reduce (0) | √ | √ | √ |  | √ |
|  | Slightly reduced (1) |  |  |  | √ |  |
|  | Reduced (2) |  |  |  |  |  |
|  | Severely reduced (3) |  |  |  |  |  |
| Total score |  | 0 | 0 | 0 | 1 | 0 |

**Day 3 post infection**

| **Clinical symptoms** | | **Mouse 1** | **Mouse 2** | **Mouse 3** | **Mouse 4** | **Mouse 5** |
| --- | --- | --- | --- | --- | --- | --- |
| rough hair | Absent (0) |  |  |  |  |  |
|  | Slightly (1) | √ | √ | √ | √ | √ |
|  | Markedly (2) |  |  |  |  |  |
| activity | Normal (0) |  |  |  |  |  |
|  | Slightly reduced (1) | √ | √ | √ |  |  |
|  | Reduced (2) |  |  |  | √ | √ |
|  | Severely reduced (3) |  |  |  |  |  |
| eye discharge | Absent (0) |  |  |  |  |  |
|  | Slightly (1) | √ | √ | √ | √ | √ |
|  | Moderate (2) |  |  |  |  |  |
|  | Severe (3) |  |  |  |  |  |
| body weight | No reduce (0) |  |  |  |  |  |
|  | Slightly reduced (1) | √ |  | √ |  |  |
|  | Reduced (2) |  | √ |  | √ |  |
|  | Severely reduced (3) |  |  |  |  | √ |
| Total score |  | 4 | 5 | 4 | 6 | 7 |

**Day 3.5 post infection**

| **Clinical symptoms** | | **Mouse 1** | **Mouse 2** | **Mouse 3** | **Mouse 4** | **Mouse 5** |
| --- | --- | --- | --- | --- | --- | --- |
| rough hair | Absent (0) |  |  |  |  |  |
|  | Slightly (1) | √ | √ | √ | √ | √ |
|  | Markedly (2) |  |  |  |  |  |
| activity | Normal (0) |  |  |  |  |  |
|  | Slightly reduced (1) | √ |  | √ |  |  |
|  | Reduced (2) |  | √ |  | √ |  |
|  | Severely reduced (3) |  |  |  |  | √ |
| eye discharge | Absent (0) |  |  |  |  |  |
|  | Slightly (1) | √ | √ | √ | √ | √ |
|  | Moderate (2) |  |  |  |  |  |
|  | Severe (3) |  |  |  |  |  |
| body weight | No reduce (0) |  |  |  |  |  |
|  | Slightly reduced (1) |  |  | √ |  |  |
|  | Reduced (2) | √ | √ |  | √ |  |
|  | Severely reduced (3) |  |  |  |  | √ |
| Total score |  | 5 | 6 | 4 | 6 | 8 |

**Day 4 post infection**

| **Clinical symptoms** | | **Mouse 1** | **Mouse 3** | **Mouse 4** |
| --- | --- | --- | --- | --- |
| rough hair | Absent (0) |  |  |  |
|  | Slightly (1) | √ | √ | √ |
|  | Markedly (2) |  |  |  |
| activity | Normal (0) |  |  |  |
|  | Slightly reduced (1) |  |  |  |
|  | Reduced (2) |  | √ | √ |
|  | Severely reduced (3) | √ |  |  |
| eye discharge | Absent (0) |  |  |  |
|  | Slightly (1) | √ | √ | √ |
|  | Moderate (2) |  |  |  |
|  | Severe (3) |  |  |  |
| body weight | No reduce (0) |  |  |  |
|  | Slightly reduced (1) |  |  |  |
|  | Reduced (2) |  |  | √ |
|  | Severely reduced (3) | √ | √ |  |
| Total score |  | 8 | 7 | 6 |

**Day 4.5 post infection**

| **Clinical symptoms** | | **Mouse 3** |
| --- | --- | --- |
| rough hair | Absent (0) |  |
|  | Slightly (1) | √ |
|  | Markedly (2) |  |
| activity | Normal (0) |  |
|  | Slightly reduced (1) |  |
|  | Reduced (2) |  |
|  | Severely reduced (3) | √ |
| eye discharge | Absent (0) |  |
|  | Slightly (1) | √ |
|  | Moderate (2) |  |
|  | Severe (3) |  |
| body weight | No reduce (0) |  |
|  | Slightly reduced (1) |  |
|  | Reduced (2) |  |
|  | Severely reduced (3) | √ |
| Total score |  | 8 |
